# Detection and Molecular Characterization of a Novel Species of Circovirus in a Tawny Owl (*Strix aluco*) in Southern Italy

**DOI:** 10.3390/ani12020135

**Published:** 2022-01-07

**Authors:** Matteo Legnardi, Laura Grassi, Giovanni Franzo, Maria Luisa Menandro, Claudia Maria Tucciarone, Adriano Minichino, Ludovico Dipineto, Luca Borrelli, Alessandro Fioretti, Mattia Cecchinato

**Affiliations:** 1 Dipartimento di Medicina Animale, Produzione e Salute, Università di Padova, Viale dell’Università 16, 35020 Legnaro, Italy; laura.grassi.2@phd.unipd.it (L.G.); giovanni.franzo@unipd.it (G.F.); marialuisa.menandro@unipd.it (M.L.M.); claudiamaria.tucciarone@unipd.it (C.M.T.); mattia.cecchinato@unipd.it (M.C.); 2 Dipartimento di Medicina Veterinaria e Produzioni Animali, Università di Napoli Federico II, Via F. Delpino 1, 80137 Napoli, Italy; adriano.minichino@gmail.com (A.M.); ludovico.dipineto@unina.it (L.D.); luca.borrelli@unina.it (L.B.); alessandro.fioretti@unina.it (A.F.)

**Keywords:** circovirus, birds of prey, tawny owl, molecular characterization, CRESS DNA viruses, wildlife, phylogenesis

## Abstract

**Simple Summary:**

The genus *Circovirus* groups some of the smallest viruses capable of autonomous replication, including some notable swine and avian pathogens. Among domestic and wild birds, circoviruses are often associated with immunosuppression and integumental disorders, but, despite their relevance, nothing is known about their circulation in birds of prey. By conducting molecular analyses on samples from birds of prey recovered by a wildlife rescue centre in Italy, we identified a new viral species in the spleen of a tawny owl (*Strix aluco*). However, there is contrasting evidence regarding its definitive host. On one hand, the virus was discovered to be phylogenetically closer to mammalian rather than avian circoviruses, which allows speculations on its host being a micromammal preyed by the tawny owl, rather than the bird itself. On the other hand, its detection in the spleen, a lymphoid organ in which other avian circoviruses are often detected, supports the tawny owl being its actual host, perhaps following a spillover event associated with predation. Adding to the growing number of circoviruses found in recent years in a diverse range of hosts, this discovery represents another step forward in the characterization of this genus of remarkable veterinary importance.

**Abstract:**

Thanks to recent developments in molecular methods, many new species have been discovered within the genus *Circovirus*, which comprises viruses of veterinary relevance found in a broad range of hosts. In particular, several circoviruses are known to infect birds, often causing immunosuppression and feathering disorders. Nonetheless, nothing is known about their circulation in birds of prey. In this study, samples from 61 birds of prey representing ten different species, recovered by a wildlife rescue centre in Southern Italy, were taken at necropsy and analysed by PCR with pan-*Circovirus* primers. Only one sample, collected from a tawny owl (*Strix aluco*), tested positive. Its genome, sequenced by primer walking, displays the typical features of circoviruses. Based on demarcation criteria, the detected strain qualifies as a novel species, which was named “tawny owl-associated circovirus” (ToCV). Phylogenetically, ToCV clustered with mammalian rather than avian circoviruses, and its closeness to a rodent circovirus suggests that its host may have been a micromammal eaten by the tawny owl. On the other hand, its detection in the spleen fits with the tropism of other avian circoviruses. Little can be therefore said on its biology and pathogenicity, and further efforts are needed to better characterize its epidemiology.

## 1. Introduction

In recent years, the advancements in molecular techniques have revolutionized the field of virology, offering increasingly available, rapidly improving high-throughput means to study viral diversity [1]. As a result, the number of newly described viral species has seen a notable surge, culminating with more than 1000 new species being officially recognized by the International Committee on Taxonomy of Viruses (ICTV) in 2020 alone [2]. In particular, a growing awareness has developed of the ubiquity of viruses with circular Rep-encoding single-stranded (CRESS) DNA genomes. These viruses, which constitute most of the ssDNA viruses, are characterized by a replication mechanism called rolling circle replication (RCR), relying on a conserved replicase [3]. Once thought to be relatively rare, CRESS DNA viruses have now been described in hosts across all domains of life by the use of metagenomics [4,5].

The taxonomical changes that reshaped the family *Circoviridae* in the last 15 years are the perfect example of the consequences of these rapid developments. The viruses belonging to this family have small, covalently closed circular genomes and are considered among the smallest viruses capable of autonomous replication in eukaryotic cells [6]. In 2000, only three species of *Circoviridae* were recognized [7], which became 12 by 2009 [8]. Today, 101 different species are known, infecting a broad range of hosts [9]. The insights gained from these discoveries led to the reassignment of the genus *Gyrovirus* from the family *Circoviridae* to *Anelloviridae*; in parallel, a new genus named *Cyclovirus* was established within *Circoviridae* [10]. A little more than a decade after the discovery of the first cyclovirus [11], more than 50 species have been described within this genus by using sequencing techniques [9]. On the other hand, little is known about their definitive hosts and biology, mostly due to their cultivation being extremely difficult [12,13].

In this timespan, the sole constant within *Circoviridae* has been the genus *Circovirus*, which includes notable veterinary pathogens such as porcine circovirus 2 (PCV-2) and beak and feather disease virus (BFDV), affecting suid and avian species, respectively. However, even this genus has undergone significant changes, with 30 out of 49 species described in the last 5 years [9]. Coherently with their small size, circoviruses display high mutation rates [14], and so far, they have been detected in a range of mammals (including cetaceans and humans) and birds, along with fishes and biting insects.

Among avian hosts, circoviruses are characterized by a tropism for epithelial and lymphatic tissues [15]. This often leads to symptoms such as beak and feather abnormalities, most notably in the case of BFDV and raven circovirus, and immunosuppression, which is also encountered with circoviruses of canaries, pigeons, ducks, zebra finches and gooses [12,16,17,18,19,20]. Despite circoviruses being detected in many avian species, including several wild birds, nothing is known about their circulation in birds of prey. As predators, these animals represent a crucial niche to investigate, since they may be exposed to a broad range of viruses [21], not limited to avian ones. The aim of this study was therefore to evaluate the presence of circoviruses in birds of prey, by considering specimens of different species from the area of Southern Italy.

## 2. Materials and Methods

### 2.1. Sampling

The sampling activities were conducted at the Wildlife Rescue Centre (CRAS) “Federico II” of the University of Naples, on rescued birds of prey that died during rehabilitation in 2019. During the routine anatomopathological examination, liver samples were collected from a total of 61 birds of prey, mostly dead due to traumatic injuries. Ten species, both diurnal and nocturnal, from three different orders were represented, including: barn owl (*Tyto alba*, n = 8), common buzzard (*Buteo buteo*, n = 2), Eurasian hobby (*Falco subbuteo*, n = 1), goshawk (*Accipiter gentilis*, n = 1), kestrel (*Falco tinnunculus*, n = 18), little owl (*Athene noctua*, n = 16), long-eared owl (*Asio otus*, n = 4), peregrine falcon (*Falco peregrinus*, n = 4), scops owl (*Otus scopus*, n = 5), tawny owl (*Strix aluco*, n = 2). Spleen samples were also collected from 13 of these birds, including two tawny owls, one long-eared owl, one goshawk, three barn owls, two peregrine falcons, two kestrels, one Eurasian hobby and one common buzzard. The choice of which matrixes to sample was based on several avian circoviruses having been detected in these sites [10]. Anamnestic details were also recorded. Samples were kept at −80 °C until processing.

### 2.2. PCR Assays

Tissue samples were eluted into PBS, then viral DNA was extracted using the DNeasy Blood & Tissue kit (QIAGEN, Hilden, Germania) following manufacturer’s instructions. The Platinum™ II Hot-Start PCR Master Mix kit (ThermoFisher, Waltham, MA, USA) was used for PCR analyses. Firstly, an assay was conducted with a pair of degenerate primers, CV1F (5′-GGIAYICCICAYYTICARGG-3′) and CV1R (5′-AWCCAICCRTARAARTCRTC-3′), designed by Li et al. [11] based on the sequences of all circoviruses known at the time. An isolate of BFDV was used as positive control. Subsequently, full genome Sanger sequencing was attempted on positive samples by primer walking. The obtained chromatograms were visually inspected and trimmed in 4Peaks (Nucleobytes B.V., Aalsmer, The Netherlands), then a consensus sequence was built on ChromasPRO (Technelysium Pty Ltd., Helensvale, QLD, Australia).

### 2.3. Phylogenetic and Genomic Analyses

Following a preliminary BLAST search [22], full genomes were aligned to a dataset including reference sequences of all currently recognised *Circovirus* species [9] using the MUSCLE algorithm [23] implemented in MegaX [24]. Phylogenetic analyses were then conducted in MegaX and SDT 1.2 [25] to assess whether the sequenced strains qualified as new viral species and to assess their relationships with other circoviruses. The ORF Finder online tool [26] was used to search for open reading frames (ORFs).

## 3. Results

### 3.1. PCR Assays

All samples but one tested negative to the PCR analysis. The positive sample was collected from the spleen of a tawny owl, while the liver sample from the same animal was negative. Based on the partial sequence amplified with the pan-*Circovirus* assay, another pair of primers, RC-F (5′-ACACCCACGTTCCGTAAAAC-3′) e RC-R (5′-CGAGAAGACCGAAGTCTTGG-3′), was designed, allowing for full genome sequencing. The genome, whose length was equal to 1745 nucleotides, was deposited in GenBank with accession number OL411978.

### 3.2. Phylogenetic and Genomic Analyses

**Figure 1:**
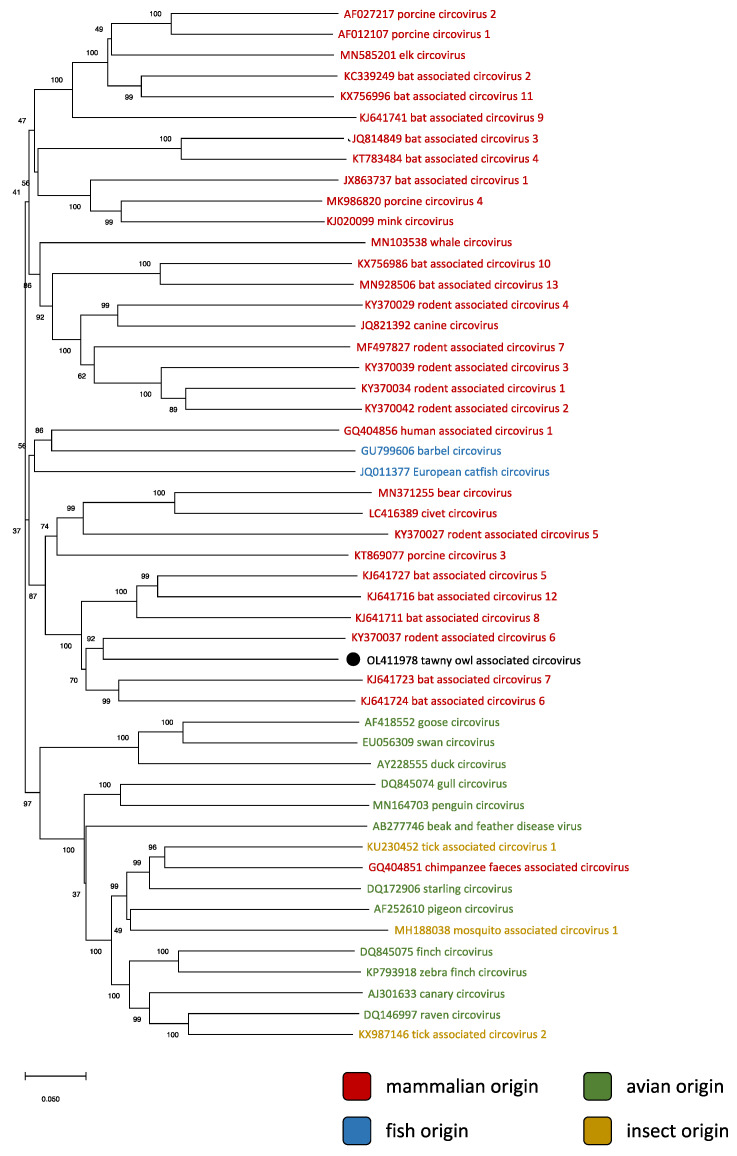
Phylogenetic relationships between the tawny owl-associated circovirus (marked with a black dot) and representatives of every known species of circovirus, whose names were color-coded based on origin. The tree was inferred using the Neighbor-Joining method [27] with pairwise deletion. The percentage of replicate trees in which the associated taxa clustered together in the bootstrap test (1000 replicates) are shown next to the branches [28].

**Figure 2:**
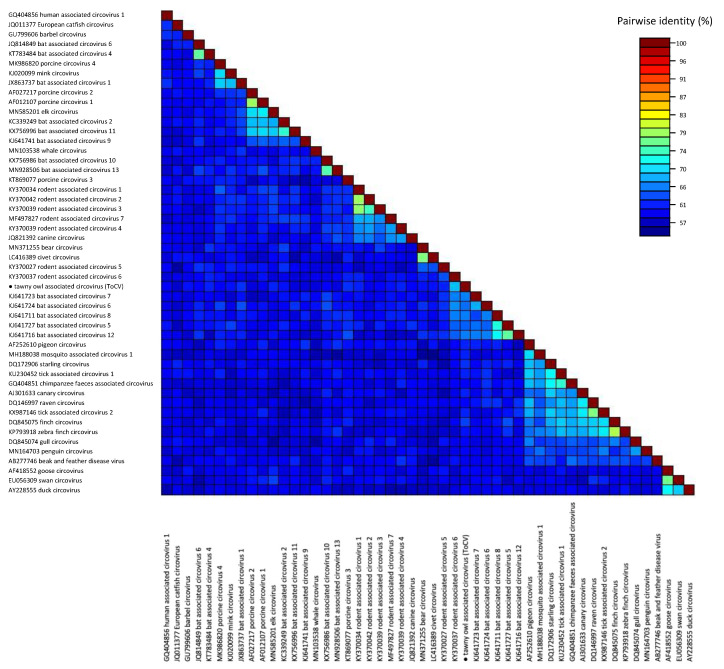
Full genome pairwise identity matrix of representative sequences of every known species of the genus *Circovirus*. The tawny owl-associated circovirus is marked with a black dot.

The phylogenetic relationships of the detected strain with representatives of currently recognized species of the genus *Circovirus* are shown in Figure 1. As revealed by the pairwise identity matrix (Figure 2), the sequenced genome showed the highest identity (69.7%) with rodent-associated circovirus 6. Therefore, according to the species demarcation threshold for the genus *Circovirus*, which is set at 80% genome-wide nucleotide pairwise identity [10], the detected virus belongs to a new species, which was tentatively named “tawny owl-associated circovirus” (ToCV).

At least two major ORFs were identified, including an 885-nt-long one (nt 164–1048, starting from the origin of replication), located on the virion strand, and a 645-nt-long one (nt 1728–1084), on the complementary strand of the replicative form. Based on analogy with the ORFs of other circoviruses, they were recognized as the *rep* and *cap* genes, respectively.

## 4. Discussion

The herein described survey made it possible to confirm the presence of circoviruses in birds of prey, albeit in only one of the 61 screened animals. The positive sample was collected from the spleen of a tawny owl that died shortly after its retrieval following a traumatic event. Besides a severe lesion on the right forearm, other anatomopathological findings included malnutrition (BCS 1), cerebellar hemorrhage, hypotrophic spleen and liver, hepatic steatosis, right atrial enlargement, and ulcers in the glandular stomach. The hematologic exam revealed a lymphocytosis with reactive heterophils. Despite the frequent association of avian circoviruses with immunosuppression and integumental diseases, it should be stressed that the sole molecular identification of ToCV does not make it possible to infer its pathogenicity, whose elucidation would require specific studies conducted on the isolated virus [29]. In addition, the reported pathological findings obviously reflect the critical condition in which the animal was found.

**Figure 3:**
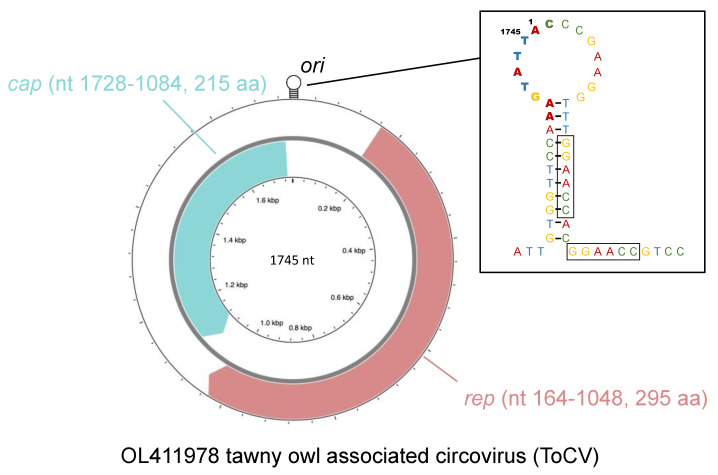
Scheme illustrating the main features of the genome of tawny owl-associated circovirus, generated with CGView Server [33], complemented with a representation of the stem-loop structure. The nonanucleotide constituting the origin of replication (*ori*) is represented in bold, while the tandemly repeated hexamers are boxed.

As for the analysis of the genomic structure, the short, circular genome displays all the typical features of circoviruses, most notably the ambisense organization and the presence of two major ORFs in different strands (Figure 3). The *rep* gene, located on the virion strand, codes for the replicase-associated protein (Rep), which plays a pivotal role in the RCR mechanism [14]. This protein is fairly conserved among all circoviruses and contains two distinct functional domains. The first, found in the N-terminal region, is a HUH (His-hydrophobe-His) endonuclease domain [30], characterized by the presence of RCR motifs I (whose sequence, in the case of ToCV, is ‘FTVNN’), II (‘PHLQG’) and III (‘YCSK’) [31]. The second, located toward the C-terminus, is a superfamily 3 (SF3) helicase domain [32] which also features three conserved motifs, named Walker-A (GPPGVGKT), Walker-B (IFDDF) and motif C (ITSN) [31].

The *cap* gene, located in the complementary strand, codes for the capsid protein (Cap), which is the sole component of the icosahedral capsid shell [34]. Despite being more heterogeneous than Rep, some conserved traits are still distinguishable, such as the accumulation of basic residues (particularly arginine) in the N-terminal region [35]. This clustering, common to all circoviruses, is thought to produce nuclear localization signal (NLS) motifs involved in nuclear localization [36]. In addition, arginine-rich motifs (ARM) also contain domains facilitating DNA binding, and the N-terminal region forms an ARM domain regulating genome encapsidation [14].

The two ORFs are separated by two intergenic regions (IRs). The largest of the two, located between the initiation codons, contains a stem–loop structure with a conserved nonanucleotide motif at its apex. This motif, which in the case of ToCV is “AAGTATTAC”, constitutes the origin of replication (*ori*), where RCR is initiated by the Rep nicking the virion strand between position 7 and 8 [37]. Additionally, a tandemly repeated hexamer “GGAACC” was found immediately to the right of the *ori*. Similar repeated sequences were identified in many other circoviruses, and are hypothesized to serve as binding sites for the Rep [38,39,40].

Based on the species demarcation criteria established for the genus *Circovirus* by Rosario et al. [10], the detected virus was established to belong to a new species, which was named “tawny owl-associated circovirus”. According to the official naming guidelines, the word “associated” should be included in the name of new circoviral species in absence of strong biological evidence identifying the definitive host [10]. This is clearly the case with ToCV, which was found to be more phylogenetically close to mammalian circoviruses than to avian ones, not only based on the whole genome, but also when considering the *rep* and *cap* sequences independently (Table 1). 

This somewhat represents an exception in the genus *Circovirus*, as species detected in mammals clearly formed a well-defined cluster, separate from the one grouping all avian circoviruses. Aside from two species found in fishes (close to mammalian circoviruses) and three detected in biting insects (located within the avian cluster), the sole exception is represented by a strain found in the stool of a chimpanzee, which clustered with viruses found in birds (Figure 1). However, the fecal origin of said sample [11] does not make it possible to confidently infer its actual host. Defining the host of ToCV therefore presents some unique challenges. 

The virus with the highest homology to ToCV is rodent-associated circovirus 6, detected in 2015 in a South China field mouse (*Apodemus draco*) [41]. This finding suggests a series of different hypotheses: one of these is the host of ToCV being a micromammal eaten by the tawny owl, rather than the bird itself. This may have happened either in the wild or during the rehabilitation period, during which the tawny owl was fed with carcasses of farmed rodents. While this scenario is extremely likely in viruses identified from stools, the detection of ToCV in the spleen but not in the liver of the same animal seems to conflict with this theory and rises some questions on the mechanism by which the virus may have passed from the enteric tract to this organ. 

The tawny owl being the actual definitive host of ToCV, perhaps as a result of a spillover from preyed mammals, would offer a much simpler explanation, as the spleen has been established as a preferential site for the detection of several avian circoviruses [10,12,42]. Duck circovirus, in particular, has been reported to reach a higher load in the spleen than in any other district, including other lymphoid organs representing major targets for a virus with well-known immunosuppressive effects [19]. Further studies will be required to establish whether this detection resulted from a sporadic spillover leading to an epidemiological dead-end or from an actual and long-lasting host jump, causing the establishment of an independent and persistent transmission cycle. The high genetic distance compared to other circoviruses might support an ancient speciation followed by independent evolution and host adaptation. Nonetheless, it is not possible to exclude other strains related to the one detected in the present study circulating in mammalian species hunted by birds of prey, and the apparently low prevalence in the considered population of birds of prey could also suggest a “contamination”, rather than the genesis of a new species. In this regard, it is worth noting that previous studies have demonstrated the possibility of foreign ingested DNA being transported through the intestinal wall and Peyer’s patches to be found in peripheral blood leukocytes and several organs, including the spleen, covalently linked to the genome [43]. Therefore, the detection of viral DNA in the spleen would not exclude a priori that the viral presence could be due to the ingestion of contaminated food, in absence of viral replication in the tawny owl. Based on this conflicting evidence, the search for the definitive host of ToCV cannot be considered concluded.

## 5. Conclusions

Even in the era of metagenomics, in which new viruses can be potentially found in every environmental sample, the discovery of a novel viral species is fascinating and may contribute to corroborate and expand our knowledge of viral ecology. The detection of ToCV, however, poses more questions than answers. So far, the phylogenetic relationships between members of the genus *Circovirus* were considered highly indicative of their definitive host. The case of ToCV seems more intricate, due to the apparent conflict between its phylogenetic closeness to mammalian circoviruses and its detection in an avian species.

Currently, different scenarios could be suggested, all starting from an original predation event: (1) spleen contamination from the alimentary tract, through the passage of non-replicating viral DNA; (2) sporadic spill-over, leading to limited replication and epidemiological dead end; (3) the viral establishment in a new host species, followed by independent evolution. Further efforts are therefore needed to shed light on its host range and epidemiology. Future investigations should be conducted not only in populations of birds of prey, but also in both wild and farmed rodents. An attempt should also be made at virus isolation on suitable cell lines, which would allow assessing its pathogenicity. In addition, the ongoing research for novel species will surely help to better define the ecology of circoviruses and understand how to better cope with a genus of such remarkable importance for veterinary infectiology.

## Figures and Tables

**Table 1:** List of the five species closest to ToCV at *rep* and *cap* level, along with the closest avian circovirus. The pairwise p-distance estimation was conducted after aligning the *rep* and *cap* sequences of the 50 representatives of the genus *Circovirus* at codon level with the MUSCLE algorithm.

*rep*		*cap*	
p-Distance	Species	p-Distance	Species
0.270	KJ370037 rodent associated circovirus 6	0.400	KJ641716 bat associated circovirus 12
0.361	KJ641727 bat associated circovirus 5	0.422	KY370037 rodent associated circovirus 6
0.363	KJ641723 bat associated circovirus 7	0.422	KJ641724 bat associated circovirus 6
0.365	KJ641711 bat associated circovirus 8	0.437	JQ814849 bat associated circovirus 3
0.373	KJ641724 bat associated circovirus 6	0.437	KX756996 bat associated circovirus 11
0.438	AB277746 beak and feather disease virus	0.489	AY228555 duck circovirus

## Data Availability

The data presented in this study are available on request from the corresponding author.

## References

[1] Plyusnin I., Kant R., Jääskeläinen A.J., Sironen T., Holm L., Vapalahti O., Smura T. (2020). Novel NGS pipeline for virus discovery from a wide spectrum of hosts and sample types. Virus Evol..

[2] Dance A. (2021). Beyond coronavirus: The virus discoveries transforming biology. Nature.

[3] Zhao L., Rosario K., Breitbart M., Duffy S. (2019). Eukaryotic Circular Rep-Encoding Single-Stranded DNA (CRESS DNA) Viruses: Ubiquitous Viruses with Small Genomes and a Diverse Host Range. Adv. Virus. Res..

[4] Shulman L., Davidson I. (2017). Viruses with Circular Single-Stranded DNA Genomes Are Everywhere!. Annu. Rev. Virol..

[5] Kazlauskas D., Varsani A., Koonin E.V., Krupovic M. (2019). Multiple origins of prokaryotic and eukaryotic single-stranded DNA viruses from bacterial and archaeal plasmids. Nat. Commun..

[6] Rosario K., Duffy S., Breitbart M. (2009). Diverse circovirus-like genome architectures revealed by environmental metagenomics. J. Gen. Virol..

[7] Van Regenmortel M.H.V., Fauquet C.M., Bishop D.H.L., Carstens E.B., Estes M.K., Lemon S.M., Maniloff J., Mayo M.A., McGeoch D.J., Pringle C.R. (2000). Virus Taxonomy. Seventh Report of the International Committee on Taxonomy of Viruses.

[8] Biagini P., Bendinelli M., Hino S., Kakkola L., Mankertz A., Niel C., Okamoto H., Raidal S., Teo C.G., Todd D., King A.M.Q., Adams M.J., Carstens E.B., Lefkowitz E.J. (2012). Family Circoviridae. Virus Taxonomy: Classification and Nomenclature of Viruses: Ninth Report of the International Committee on Taxonomy of Viruses.

[9] Virus Taxonomy. https://talk.ictvonline.org/ictv-reports.

[10] Rosario K., Breitbart M., Harrach B., Segalés J., Delwart E., Biagini P., Varsani A. (2017). Revisiting the taxonomy of the family Circoviridae: Establishment of the genus Cyclovirus and removal of the genus Gyrovirus. Arch. Virol..

[11] Li L., Kapoor A., Slikas B., Bamidele O.S., Wang C., Shaukat S., Alam Masroor M., Wilson M.L., Ndjango J.-B.N., Peeters M. (2010). Multiple diverse circoviruses infect farm animals and are commonly found in human and chimpanzee feces. J. Virol..

[12] Todd D. (2000). Circoviruses: Immunosuppressive threats to avian species: A review. Avian Pathol..

[13] Cruz T.F., Araujo J.P. (2014). Cultivation of PCV2 in swine testicle cells using the shell vial technique and monitoring of viral replication by qPCR and RT-qPCR. J. Virol. Methods.

[14] Nath B.K., Das S., Roby J.A., Sarker S., Luque D., Raidal S.R., Forwood J.K. (2021). Structural Perspectives of Beak and Feather Disease Virus and Porcine Circovirus Proteins. Viral Immunol..

[15] Todd D., Gortázar C., Gavier-Widén D., Duff P.J., Meredith A. (2012). Circovirus Infections. Infectious Diseases of Wild Mammals and Birds in Europe.

[16] Todd D. (2004). Avian circovirus diseases: Lessons for the study of PMWS. Vet. Microbiol..

[17] Stewart M.E., Perry R., Raidal S.R. (2006). Identification of a novel circovirus in Australian ravens (Corvus coronoides) with feather disease. Avian Pathol..

[18] Rinder M., Schmitz A., Peschel A., Wörle B., Gerlach H., Korbel R. (2017). Molecular characterization of a recently identified circovirus in zebra finches (Taeniopygia guttata) associated with immunosuppression and opportunistic infections. Avian Pathol..

[19] Hong Y.T., Kang M., Jang H.K. (2018). Pathogenesis of duck circovirus genotype 1 in experimentally infected Pekin ducks. Poult. Sci..

[20] Sheykhi A., Sheikhi N., Charkhkar S., Brujeni G.N. (2018). Detection and characterization of circovirus in canary flocks. Avian Dis..

[21] Malmberg J.L., White L.A., VandeWoude S. (2021). Bioaccumulation of pathogen exposure in top predators. Trends Ecol. Evol..

[22] Altschul S.F., Gish W., Miller W., Myers E.W., Lipman D.J. (1990). Basic local alignment search tool. J. Mol. Biol..

[23] Edgar R.C. (2004). MUSCLE: Multiple sequence alignment with high accuracy and high throughput. Nucleic Acids Res..

[24] Kumar S., Stecher G., Li M., Knyaz C., Tamura K. (2018). MEGA X: Molecular evolutionary genetics analysis across computing platforms. Mol. Biol. Evol..

[25] Muhire B.M., Varsani A., Martin D.P. (2014). SDT: A virus classification tool based on pairwise sequence alignment and identity calculation. PLoS ONE.

[26] ORF Finder. https://www.ncbi.nlm.nih.gov/orffinder/.

[27] Saitou N., Nei M. (1987). The neighbor-joining method: A new method for reconstructing phylogenetic trees. Mol. Biol. Evol..

[28] Felsenstein J. (1985). Confidence limits on phylogenies: An approach using the bootstrap. Evolution.

[29] dos Santos F.A., Portela S.J., Nogueira T., Carvalho C.L., de Sousa R., Duarte M.D. (2021). Harmless or Threatening? Interpreting the Results of Molecular Diagnosis in the Context of Virus-Host Relationships. Front. Microbiol..

[30] Ilyina T.V., Koonin E.V. (1992). Conserved sequence motifs in the initiator proteins for rolling circle DNA replication encoded by diverse replicons from eubacteria, eucaryotes and archaebacteria. Nucleic Acids Res..

[31] Rosario K., Duffy S., Breitbart M. (2012). A field guide to eukaryotic circular single-stranded DNA viruses: Insights gained from metagenomics. Arch. Virol..

[32] Gorbalenya A.E., Koonin E.V. (1993). Helicases: Amino acid sequence comparisons and structure-function relationships. Curr. Opin. Struct. Biol..

[33] Stothard P., Grant J.R., Van Domselaar G. (2019). Visualizing and comparing circular genomes using the CGView family of tools. Brief. Bioinform..

[34] Sarker S., Terrón M.C., Khandokar Y., Aragão D., Hardy J.M., Radjainia M., Jiménez-Zaragoza M., De Pablo P.J., Coulibaly F., Luque D. (2016). Structural insights into the assembly and regulation of distinct viral capsid complexes. Nat. Commun..

[35] Heath L., Williamson A.-L., Rybicki E.P. (2006). The Capsid Protein of Beak and Feather Disease Virus Binds to the Viral DNA and Is Responsible for Transporting the Replication-Associated Protein into the Nucleus. J. Virol..

[36] Chen J.K., Hsiao C., Lo A.R., Wang C.Y. (2020). Characterization of the nuclear localization sequence of beak and feather disease virus capsid proteins and their assembly into virus-like particles. Virus Res..

[37] Steinfeldt T., Finsterbusch T., Mankertz A. (2006). Demonstration of nicking/joining activity at the origin of dna replication associated with the rep and rep’ proteins of porcine circovirus type 1. J. Virol..

[38] Mankertz A., Hattermann K., Ehlers B., Soike D. (2000). Cloningand sequencing of columbid circovirus (CoCV), a new circovirus from pigeons. Arch. Virol..

[39] Phenix K.V., Weston J.H., Ypelaar I., Lavazza A., Smyth J.A., Todd D., Wilcox G.E., Raidal S.R. (2001). Nucleotide sequence analysis of a novel circovirus of canaries and its relationship to other members of the genus Circovirus of the family Circoviridae. J. Gen. Virol..

[40] Todd D., Weston J.H., Soike D., Smyth J.A. (2001). Genome Sequence Determinations and Analyses of Novel Circoviruses from Goose and Pigeon. Virology.

[41] Wu Z., Lu L., Du J., Yang L., Ren X., Liu B., Jiang J., Yang J., Dong J., Sun L. (2018). Comparative analysis of rodent and small mammal viromes to better understand the wildlife origin of emerging infectious diseases. Microbiome.

[42] Stenzel T., Dziewulska D., Tykałowski B., Koncicki A. (2020). The Clinical Infection with Pigeon Circovirus (PiCV) Leads to Lymphocyte B Apoptosis But Has No Effect on Lymphocyte T Subpopulation. Pathogens.

[43] Schubbert R., Renz D., Schmitz B., Doerfler W. (1997). Foreign (M13) DNA ingested by mice reaches peripheral leukocytes, spleen, and liver via the intestinal wall mucosa and can be covalently linked to mouse DNA. Proc. Natl. Acad. Sci. USA.

